# Supplementary material for: Complexome profiling on the Chlamydomonas lpa2 mutant reveals insights into PSII biogenesis and new PSII associated proteins
Source: J Exp Bot. 2021 Aug 26;73(1):245–62. doi: 10.1093/jxb/erab390 (PMC8730698; doi:10.1093/jxb/erab390)
Supplement: erab390_suppl_Supplementary_Dataset_S1 [file erab390_suppl_supplementary_dataset_s1.zip › Supplemental Dataset 1 - Excel List and all profiles/plots/GLC2A_Cre03.g190500.html]

Trivial name: GLC2A  
  
Euclidean distance: 34313.48  
Mean Intensity (WT): 958.23  
Mean Intensity (Mut): 2276.69  
Distance: 15.07  
  
MapMan: misc.gluco-, galacto- and mannosidases.alpha-galactosidase;misc.gluco-, galacto- and mannosidases;major CHO metabolism.degradation.starch.starch cleavage  
  
p value of intensity sums Welch test: 0.1479
